# Images of depression in Charles Baudelaire: clinical understanding in the context of poetry and social history

**DOI:** 10.1192/bjb.2022.84

**Published:** 2024-02

**Authors:** Giovanni Stanghellini, George Ikkos

**Affiliations:** 1 University of Florence, Italy; 2 ‘D. Portales’ University, Santiago, Chile; 3 Royal National Orthopaedic Hospital, Stanmore, UK

**Keywords:** Mood, phenomenology, images, poetry, critical theory

## Abstract

There is increasing recognition of the importance of the humanities and arts in medical and psychiatric training. We explore the poetry of Charles Baudelaire (1821–1867) and its evocations of depression through themes of mood, time and self-consciousness and discuss their relation to images of ‘spleen’, the ‘snuffling clock’ and the ‘sinister mirror’. Following the literary critical commentaries of Walter Benjamin (1892–1940) and Jean Starobinski (1920–2019) we identify some of their roots in the poet's experience of the rapid and alienating urbanisation of 19th-century Paris. Appreciation of the rich vocabulary of poetry and the images it generates adds depth to clinical practice by painting vivid pictures of subjective experience, including subjective experience of the ‘social’ as part of the biopsychosocial constellation.

‘The same song was repeated to me elsewhere; no one wanted to admit that science and poetry could be combined. It was forgotten that science came out of poetry and it was not considered that by changing the times these two could amicably find themselves with mutual advantage on a higher level’

J.W. Goethe^1^

There is increasing recognition of the importance of the humanities, including poetry,^2,3^ arts and visual images^4,5^ in psychiatric education. Here we suggest that poetic images can stimulate engagement with patients’ personal and social experience to overcome what we see as the curtailment of curiosity and impoverishment of practice that the reifying and static vocabularies of diagnostic manuals, descriptive psychopathology and individual symptom monitoring contribute to. These are reductive maps of patients’ ‘mindscapes’,^6^ i.e. their experience of their worlds (space and the objects it contains, other people, the patient's own body and passing of time). Such maps often take patients helpfully from distressed state A to the desired B. However, other times they lead in the wrong direction, for example with missed opportunities for dialogue, reflection and insight. In contrast, contemplation of mindscapes through poetic images offers clinicians the prospect of a richer, more felicitous and dynamic phenomenology and, thus, opportunities for integration and healing that reductive maps cannot.

## Mood, melancholia and society

The emergence of psychopharmacology in the 1950s reinforced the inclination to think of disturbed mental states as medical conditions.^7^ However, their protean nature has challenged the aspiration to establish natural diagnostic boundaries consistent with a biomedical model.^8^ The (only partially successful) response to this challenge has been to establish diagnoses by committee. However, the high degrees of comorbidity between mental disorders suggest that the allegedly distinct categories of DSM-5 and ICD-11 are oversimplifications. With respect to depression, an allegation is that the concept has been oversimplified and diagnosis overused for the benefit of others, more than patients.^9^ Despite assurances of evidence-based indication for antidepressant prescription^10^ concerns about the balance of benefit to harm remain, particularly from epidemiological perspectives.^11^ We must complement diagnostic and symptom monitoring practice with a more nuanced approach, including attention to the language our patients use to describe their condition, also everyday language in general use and language used in literature and the humanities.

In response to concerns about overuse of the diagnosis of depression, some^12–14^ have proposed return of focus to the historically persistent concept of ‘melancholia’, with emphasis on more severe forms of depression, hypothesised as more biologically determined. However, it has proven impossible to establish reliable diagnostic markers confirming that this category ‘carves nature at its joints’.^15,16^ Furthermore, though the term ‘melancholia’ brings the assurance of a clinical history dating back to the remote past,^17^ the discourse around it is culturally more multilayered than medical aspiration to a biological condition suggests.^18–21^ Here, therefore, we aim to illustrate the clinical relevance of this wider tradition through an important fragment of its history, the poetry of Charles Baudelaire (1821–1867) and commentaries on it by Walter Benjamin (1892–1940) and Jean Starobinski (1920–2019). We also relate these to writings on the phenomenological psychopathology of depression. Although the boundaries of the clinical phenotype of ‘melancholia’ are porous, it is the retarded type of depression that our text bears on more rather than others, though not exclusively so.

A final point before we proceed. Although criticisms of George Engel's biopsychosocial model have been made and the concept does have problems,^22^ his proposal has maintained utility^23^ precisely because of its aspiration to integrate biological and psychological factors with social contexts. This notwithstanding, in practice psychiatry's approach to ‘the social’ in the ‘biopsychosocial’ remains somewhat superficial. It is only a small exaggeration to suggest that the social in the biopsychosocial model of psychiatry has reduced the complexities of the social dimension of psychopathological conditions to aspirations of ‘objective’ generalisations about shared history, beliefs and customs of communities or such ‘accounting’ as may be found in social statistics. Perusal of social psychiatry textbooks (e.g.^24^) will find plenty of appropriate references to epidemiology and social science but limited reference to the subjective experience of the social. Yet research has underscored the importance of subjectivity to psychopathology.^25^ It is relevant therefore that Benjamin's and Starobinski's commentaries shed light on the relations between Baudelaire's subjective poetic images and their social-historical context.

## Images of depression

Baudelaire's was a time of profound social transformation when classical capitalist supremacy established the modern metropolis, liberal state and global trade associated with imperialism.^26,27^ Writing in the Paris of France's second Empire (1852–1870), in his monograph *The Painter of Modern Life*^28^ Baudelaire coined the term ‘modernity’ as the ‘ephemeral, the fugitive, the contingent’. He was simultaneously fascinated by and disapproving of modernity; according to Benjamin a ‘secret agent’^29^– both member of the bourgeoisie and critical testimony of its dissatisfaction with the power it was exercising. Melancholy was to Baudelaire the very essence of modernity *and* of poetry: a spiritual state associated with a condition, not of loss but unfulfillment.

Baudelaire's transformational collection of poems *The Flowers of Evil*^30^ was the only one published during his lifetime. Including themes of lesbian love and its consummation, recreational drugs and decay, it scandalised bourgeois society and was censored. The collection also evokes at least three sets of images which can lead to a nuanced insight into the phenomenology of depression in its social context: ‘spleen’, ‘snuffling clock’ and ‘sinister mirror’. These resonate with three theories of depression in contemporary phenomenological psychopathology: as a disorder of mood (spleen), temporality (clock) and self-consciousness (mirror).

### Spleen: depression as a disorder of mood

L'Espoir,
Vaincue, pleure, et l'Angoisse atroce, despotique,
Sur mon crane incline plante son Drapeau noire

*‘*Hope/Defeated, weeps, and the atrocious, despotic Anguish,/On my tilted skull, plants its black flag’ concludes ‘Spleen IV’ [#78]. The first way of looking at depression is as a condition characterised by a kind of mood. Moods are often ineffable phenomena hardly amenable to analysis or rational explanation. They are like an atmosphere which suffuses overall experience with an affective tinge. They colour pervasively the way we experience the world, other people and ourselves.^31^ The ‘humour’ (state of feelings or mood) characterising certain forms of depression, is called by Baudelaire ‘spleen’ – it refers to the slowing or stopping of the flow of blood and other bodily fluids and suggests associations with hypochondriacal malaise, restriction, heaviness, bitterness, rottenness. The ancient theory of temperaments postulates the spleen as the organ in which circulation slows down and blood becomes thick, cold and dry, generating the melancholia (*melaina chole*). ‘Spleen IV’ (#78) abounds with feelings of constriction, congestion and stagnation: a ‘low, heavy sky weighs like a lid’ on the groaning spirit, and the earth is changed into a ‘humid dungeon’ in which, like a bat, hope goes ‘beating the wall's with her timid wings ‘knocking her head’ against the ‘rotten ceiling’. Meanwhile, rain stretches out its endless drip like ‘bars of a vast prison’.

This poem ends with another iconic image of depression, which has been extensively documented^18^ and portrayed by Dürer in his 1514 print *Melencolia I*: the ‘bowed head’, i.e. the chin resting heavily and inert on the hand. Its iconic recurrence was captured by Baudelaire's German translator Benjamin when he wrote ‘the downward gaze is characteristic of the saturnine, who bore through the ground with their eyes’.^32^

### Snuffling clock: depression as a disorder of lived temporality

Le bourdon se lamente, et la bûche enfumée
Accompagne en fausset la pendule enrhumée

*‘*The great bell whines, the smoking log/accompanies in falsetto the snuffling clock’ is an image in ‘Spleen I’ [#75]. For Benjamin, Baudelaire's spleen is neither sadness (the feeling of sinking into sorrow after the loss of the beloved) nor melancholy (a sublime spiritual state associated with loss). It is the degradation of experience arising from massive urbanisation and commodification of relationships. Here we find a second image of depression, linked to temporality rather than to mood: *desynchronisation.* In depression time is sick: its icon can be found in the ‘snuffling clock’ – *la pendule enrhumée.* The image of *la pendule enrhumée* links time (the clock) to stagnating humours (the French noun *rhume* means cold/catarrh: ‘a build-up of mucus in an airway or cavity of the body’^33^). It encapsulates the freezing of time, a respiratory illness and the stagnation of mucus.

In ‘Spleen II’ [#76], depression is described as a lagging behind caused by a kind of lameness: ‘Nothing equals the slowness of those lame days’. Desynchronisation, according to Benjamin, is more than mere sluggishness: it is technological asynchronism. For example, in ‘The Swan’:

Old Paris is no more (the shape of a city
Changes faster, alas! than the heart of a mortal)

Benjamin's understanding of depressive desynchronisation links depression to modernity, urbanisation, the advent of artificial lighting – and finally the discord between the time of Man and that of the Cosmos, a theory also developed by phenomenological psychopathologists Tellenbach^34^ and Fuchs.^35^ In natural environments, when ‘evening twilight’ challenges vision with fading light, our senses sharpen in compensation, generating perceptual alertness and a dreamlike vision and impression of transcendent reality. However, in cities artificial illumination and its amplifying stimulation overtakes the day's slow retreat and blunts the senses. Hence, especially the atmospheric senses such as taste and smell, weaken and ‘adorable Spring has lost its scent’. Crucially all this occurs when people have come primarily to ‘know one another as creditors and debtors, as sellers and customers, as employers and employees – above all they know one another as competitors’. The resulting experience is a sense of ‘permanent catastrophe’.^36^

Jean Starobinski^37^ also discerns in Baudelaire specific aspects of the depressed person's experience at the dawn of modernity. Baudelaire was one of the first to write that being depressed means losing the feeling of correspondence between one's own inner time and the movement of external things. The depressed feels that the world is a dramatically accelerating external spectacle and, at the same time, his response is delayed by some a sort of immobilising impediment. Starobinski wonders whether the dizzying destructions and reconstructions of urbanisation of the modern era cause feelings of spleen and exile or whether Baudelaire's images are simply evoked because of the troubled mood of the melancholic who is not at peace until he can project his feelings onto an object. Which is the ‘basic’ phenomenon in depression? Is it primarily a disorder of temporality, a condition based on the desynchronisation between the personal and the city's time or is it first and foremost a disorder of mood, rooted in changing feelings in the most sensuous meaning of the word. The same dilemma in understanding depression may be found in phenomenological psychopathology. Straus^38^ and Minkowski^39^ advocate the first explanation, Binswanger^40^ the second. In our view, the images are equally valid and interrelated.

### Sinister mirror: depression as a disorder of self-consciousness.

Ne suis-je pas un faux accord
Dans la divine symphonie,
Grâce à la vorace Ironie
Qui me secoue et qui me mord?

‘Am I not a false chord/In the divine symphony,/Thanks to the voracious Irony/That scares me and bites me?’. The image of depression as someone contemplating himself in a mirror, the self-reflexive alienation of a person devoured by self-irony, can be found in ‘Eautontimorumenos’ (‘The Self-Tormenter’ [#83]. Depression is a severe kind of depersonalisation rooted in the nature of human self-consciousness which is capable of splitting between observing and observed Ego. When the split is complete, the first feels inane while contemplating the second in its despairing immutability. In ‘Beyond Redemption’ [#84] Baudelaire represents depression as a *tête-à-tête*, somber and limpid at the same time, of a heart that has become the mirror of itself, a well of truth, clear and black, where a pale star flickers – the ‘consciousness of Evil’:

I am the sinister mirror
In which the vixen looks [our translation]

For Baudelaire, because of a split of consciousness (but not due to masochism), the depressed is the ‘vampire’ of his own heart, ‘the wound and the dagger, the blow and the cheek, the members and the wheel, victim and executioner’. ‘Voracious irony’ which ‘shakes’ and ‘bites’ him, worsens depersonalisation and desynchronisation from the ‘heavenly symphony’, commanding eternal solitary sarcastic laughter and incapacity to smile sympathetically.

This understanding of depression can be found in Kraus and other clinical phenomenologists.^41,42^ What torments the severely depressed is their overwhelming sense of isolation rooted in the ‘feeling of the loss of feeling’,^43^ an alarming sense of profound indifference or emotional detachment. They lament their emotional anaesthesia, especially inability to establish relationships with and love others.^41^ Their loneliness is complemented by self-accusations and a sense of guilt. They speak of their past as characterised by inauthenticity. They compulsively criticise their previous solicitude and ‘over-involvement’ with others (i.e. the kind feelings they used to have when not in acute depressive states), which they now see as fake. They chastise their own inability to love others sincerely. Their whole experience is permeated by judgements on what is right or wrong, authentic or inauthentic, sincere or dishonest.

## Depressive transformations

Baudelaire's images can help rehumanise the condition we – the post-moderns – renamed over-inclusively as ‘depression’. They can help us relate to the patient's experience of transformation. A depressive ‘state’ is one point, the outcome of developing a depressive state – a process embedded in a specific biological, personal (emotional, cognitive and axiological) and interpersonal (relational, cultural and social) context. Baudelaire's images – spleen, the snuffling clock and the sinister mirror – are not three distinct kinds of depression, but three stages of one biopsychosocial metamorphosis. They illustrate the transformation process occurring when the conative-affective operation of vital dynamics is impaired, the temporal movement of becoming is blocked, and the self-world reciprocal accord is disrupted and desynchronised.

These images illustrate the transformation process of a liquid that slows down as its flow stagnates and the vital stream stops becoming – the sick clock. The sickening of the clock implies the spleen: a sense of sluggishness, inaction and lethargy are the main phenomenal features of the former, and localised or generalised feelings of increased rigidity of the lived body are those of the latter. The body is experienced as a heavy and rigid burden and an obstacle to spontaneous engagement in everyday life. A concurrent feature of temporal deceleration and bodily stasis is that one does not feel equal to the pace of one's social environment. In this stasis, one feels irrevocably and painfully separated from the flowing of the macro-cosmos one used to belong and be attuned to. The outcome is estrangement from the interpersonal world. Furthermore, the images of sick clock and spleen imply that of the mirror: when existence reaches the impasse of a dead-end track, it thickens, condensates and helplessly witnesses its own transformation as it mineralises into a lifeless crystal which can only reflect perpetually the sinister image of a petrified, putrefied and insulated self-tormenting Self.

## Conclusions

As it is drawn from the writings of a single person, the French poet Baudelaire, our account is not empirical in the contemporary evidence-based medical sense of the word. Nevertheless, its wider relevance is intimated by the subjective nature of feeling, the poet's personal history, his seminal position as a poet in literary history and his wide appeal across continents and centuries. Benjamin suggested that the genius of Baudelaire transformed the immediate experience (*Erlebnis*) of the daily shocks (*Chockerlebnis*) of 19th-century metropolitan capitalism into long experience (*Erfahrung*), that is experience contextualised both in space and time. In the image and words of an earlier German poet ‘it is the sea that takes and gives remembrance [ … ] and love no less keeps eyes attentively fixed’ but ‘what is lasting poets provide’.^44^ Thus perceived, the images of poetry can help us appropriate and make sense of what we have endured. Endured in both the sense of what we have suffered at certain times, and of our persistence in time.

In conclusion, without requiring universality, the wide impact and enduring appeal of poetic images, including Baudelaire's, confirm that some speak of people and their times more broadly, capturing their (social) world and (lived) history in a way that is otherwise impossible to match in force and concision. They are profoundly descriptive in a way that descriptive psychopathology can never aspire to and they convey the fulness of experience more vividly than philosophically rooted disciplines like phenomenological psychopathology. And although we do not propose our approach as an alternative to descriptive and phenomenological psychopathology, we hope these preliminary considerations will help stimulate clinicians’ interest in a different systematic investigation of experience and historicity of mental symptoms, resulting in better therapeutic rapport, more opportunities for healing and marked quality improvement in everyday practice. As Einstein remarked: ‘The value of an education in a liberal arts college is not the learning of many facts but the training of the mind to think something that cannot be learned from textbooks’.^45^

## Data Availability

Data availability is not applicable to this article as no new data were created or analysed in this study.

## About the authors

**Giovanni Stanghellini**, MD, DPhil (HC), is Full Professor of Dynamic Psychology in the Department of Health Sciences at the University of Florence, Italy, and Adjunctive Professor in ‘D. Portales’ University, Santiago, Chile. **George Ikkos**, Hon FRCPsych, FRSA, is a consultant liaison psychiatrist at the Royal National Orthopaedic Hospital, Stanmore, UK.
